# Supplementary material for: The full-length BEND2 protein is dispensable for spermatogenesis but required for setting the ovarian reserve in mice
Source: eLife. 2025 Aug 20;13:RP96052. doi: 10.7554/eLife.96052 (PMC12367297; doi:10.7554/eLife.96052)
Supplement: Figure 1—figure supplement 1—source data 2. [file elife-96052-fig1-figsupp1-data2.zip › Figure S1-source data 2/FigureS1 sourcedata2.pdf]

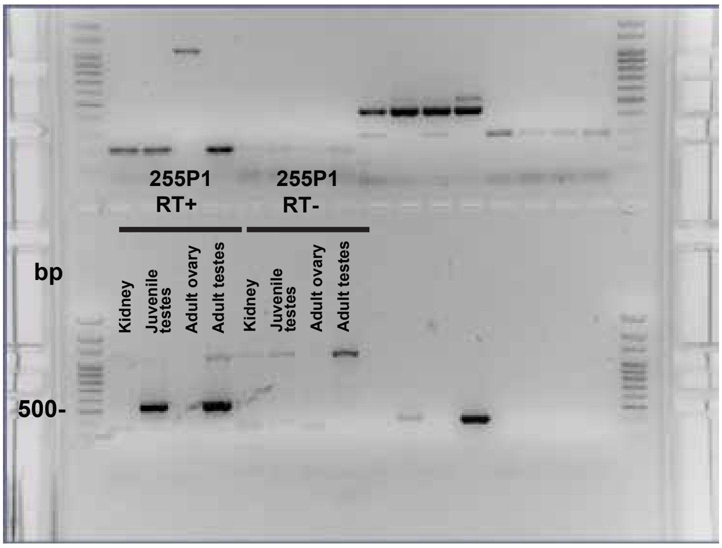

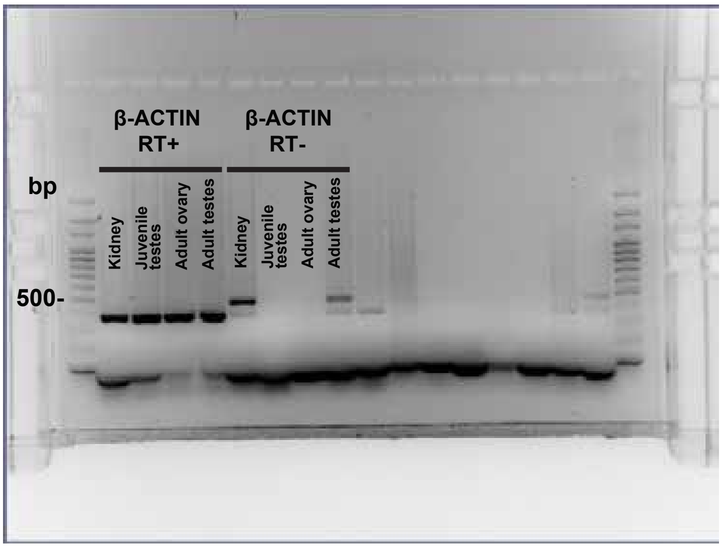

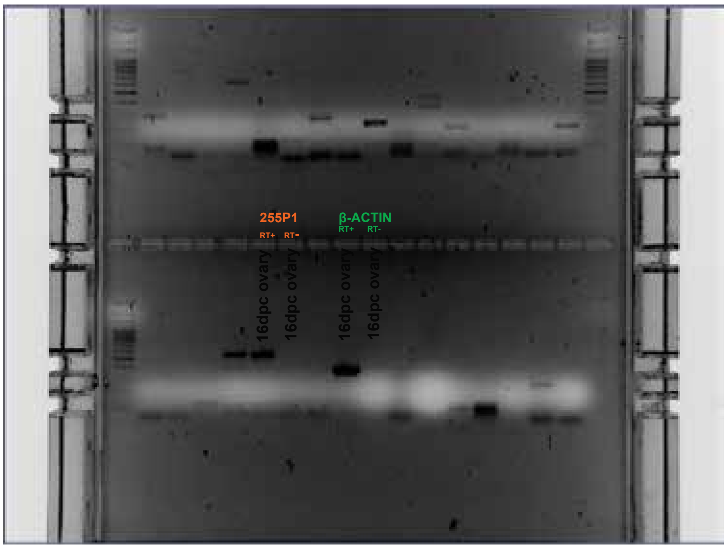

Figure S1, Source Data 2.
Original gel corresponding to Figure 1, panel A.
The labeled lanes show 255P1 expression in different mouse tissues; β-ACTIN serves as a loading control.
