# Supplementary figures and images for: The full-length BEND2 protein is dispensable for spermatogenesis but required for setting the ovarian reserve in mice
Source: eLife. 2025 Aug 20;13:RP96052. doi: 10.7554/eLife.96052 (PMC12367297; doi:10.7554/eLife.96052)

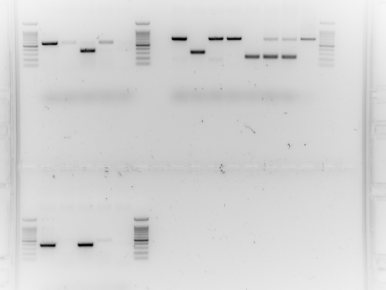

Supplement: Figure 2—source data 1. [file elife-96052-fig2-data1.zip › Figure 2-source data 1/Raw_figure2B.tif]

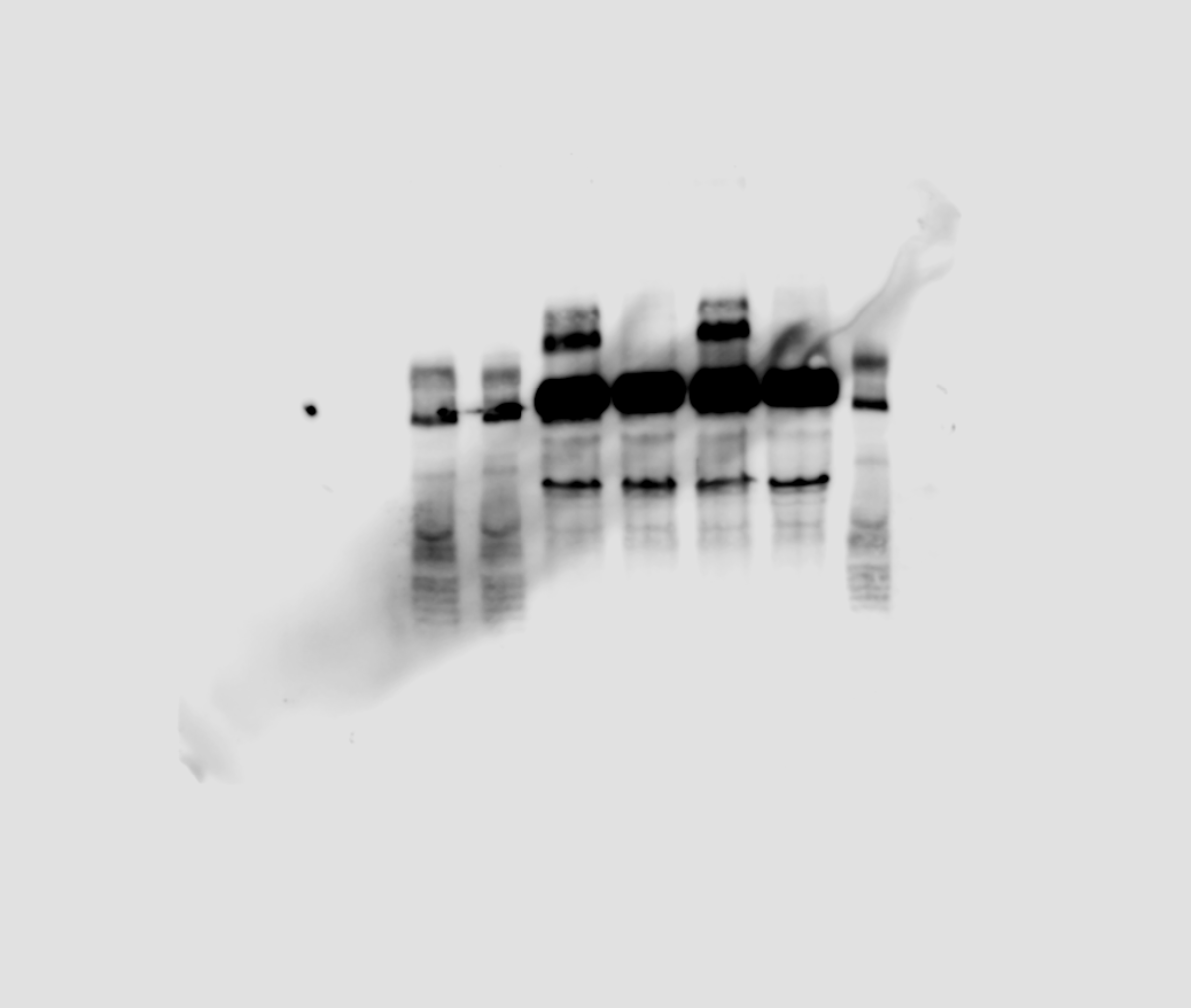

Supplement: Figure 2—source data 1. [file elife-96052-fig2-data1.zip › Figure 2-source data 1/Raw_Figure2C.png]

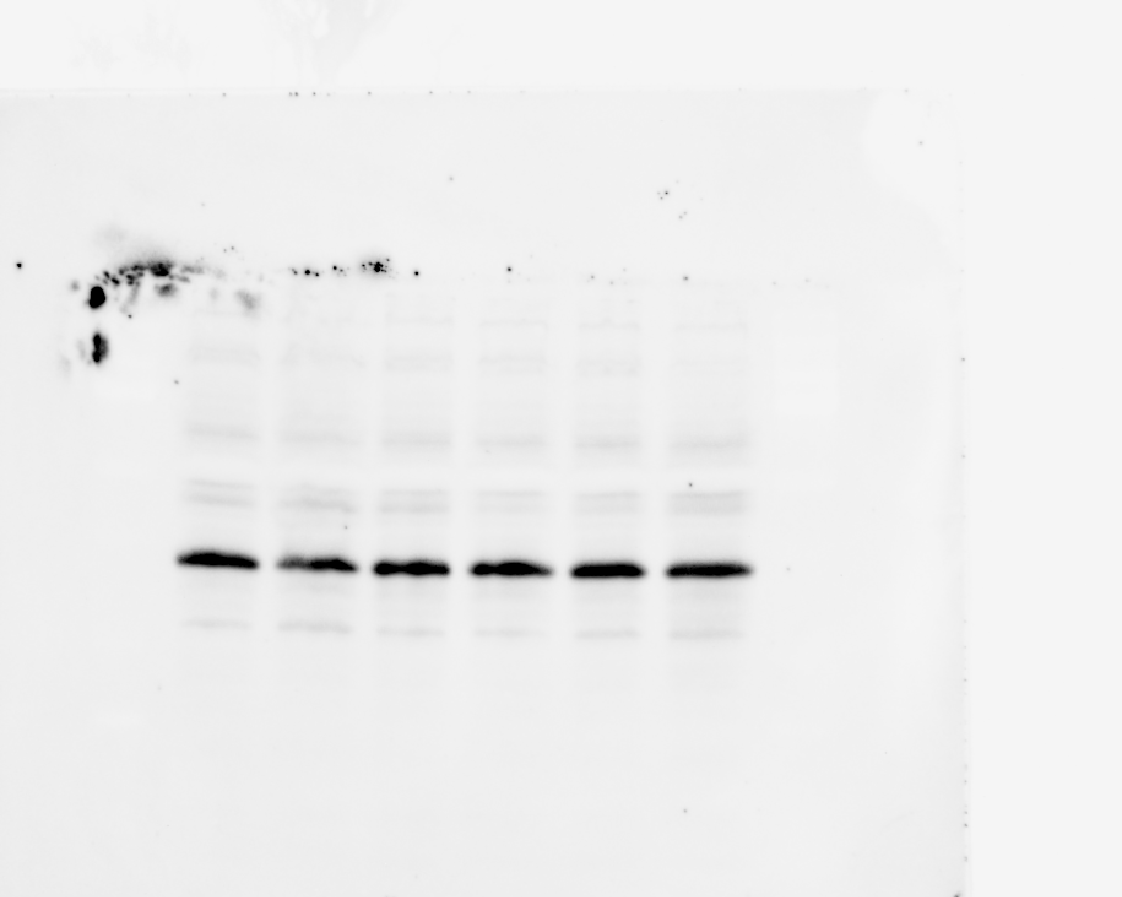

Supplement: Figure 4—figure supplement 1—source data 1. [file elife-96052-fig4-figsupp1-data1.zip › Figure S2-source data 1/Raw_FigS2D_GAPDH.tif]

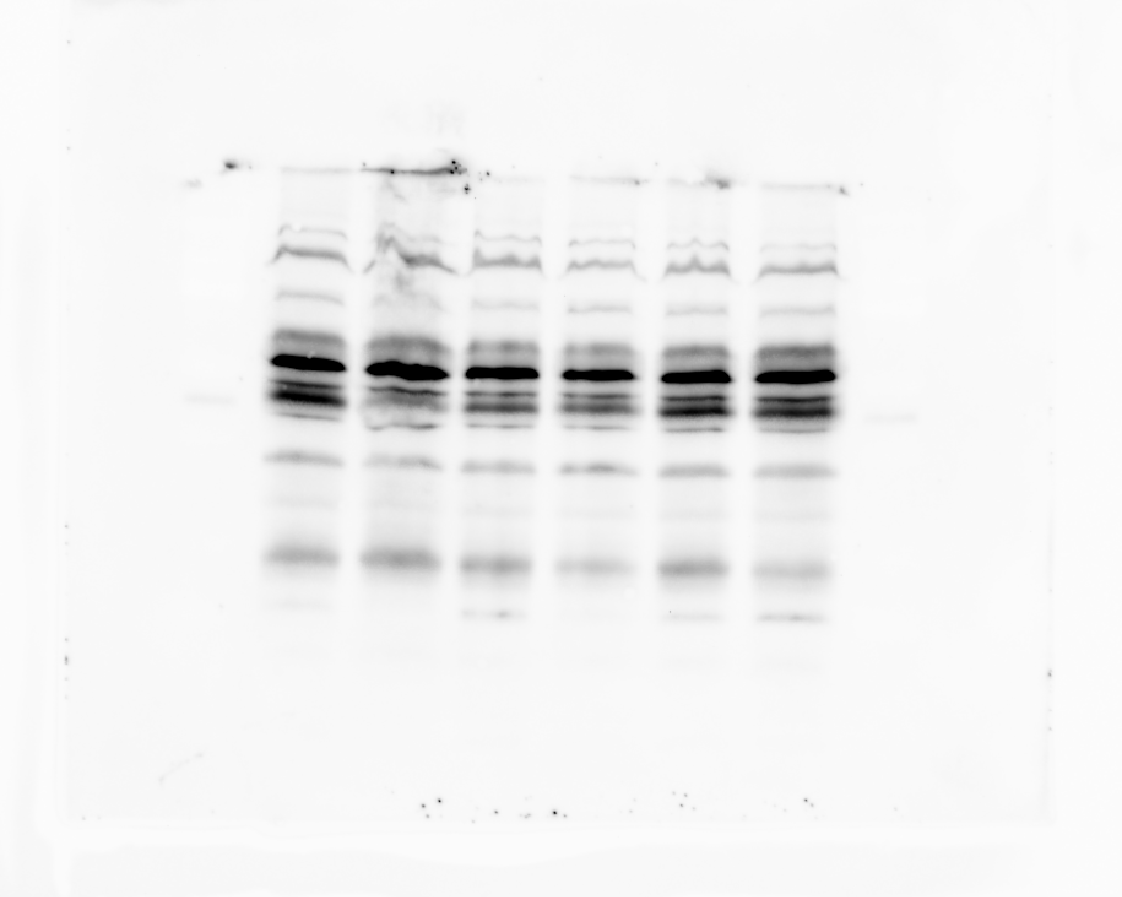

Supplement: Figure 4—figure supplement 1—source data 1. [file elife-96052-fig4-figsupp1-data1.zip › Figure S2-source data 1/Raw_FigS2D_KU70.tif]

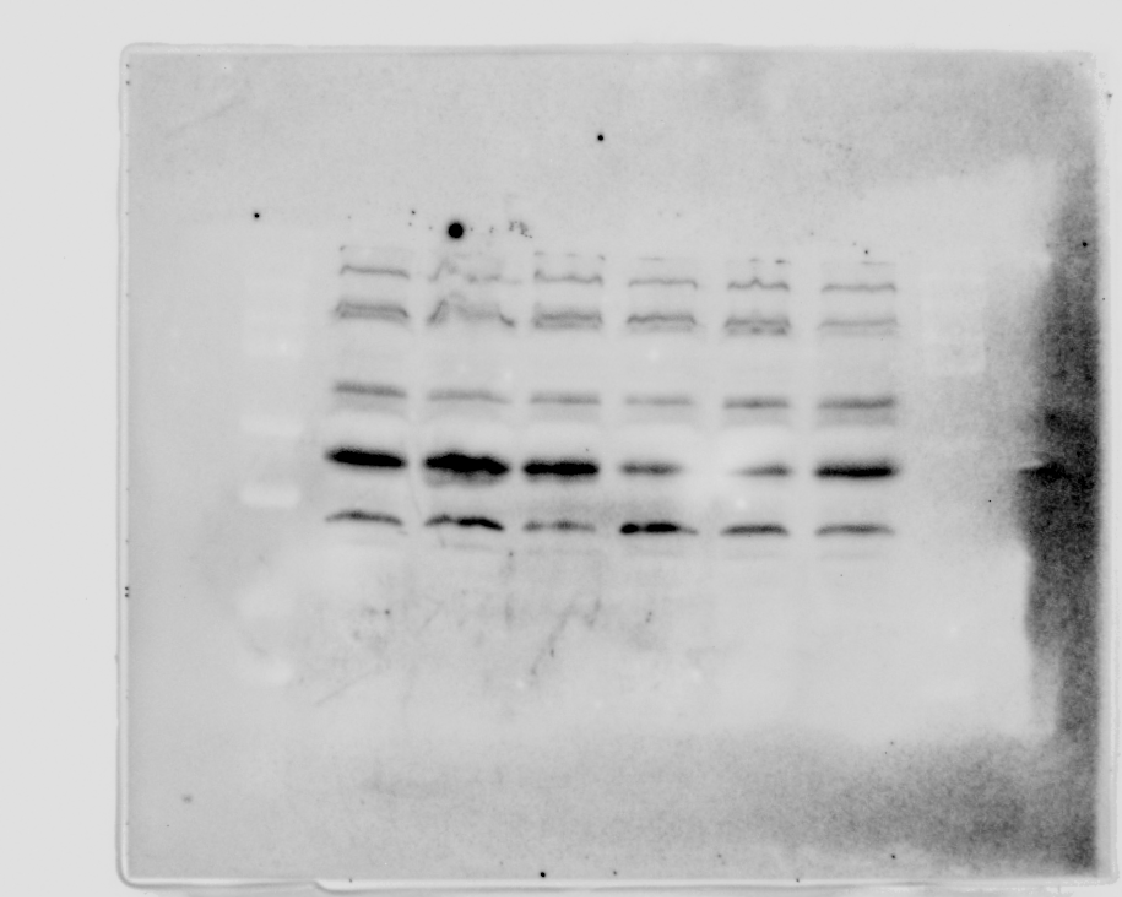

Supplement: Figure 4—figure supplement 1—source data 1. [file elife-96052-fig4-figsupp1-data1.zip › Figure S2-source data 1/Raw_FigS2D_LINE1.tif]
