# Supplementary material for: The full-length BEND2 protein is dispensable for spermatogenesis but required for setting the ovarian reserve in mice
Source: eLife. 2025 Aug 20;13:RP96052. doi: 10.7554/eLife.96052 (PMC12367297; doi:10.7554/eLife.96052)
Supplement: Figure 2—source data 2. [file elife-96052-fig2-data2.zip › Figure 2-source data 2/Figure2 sourcedata2_vor.pdf]

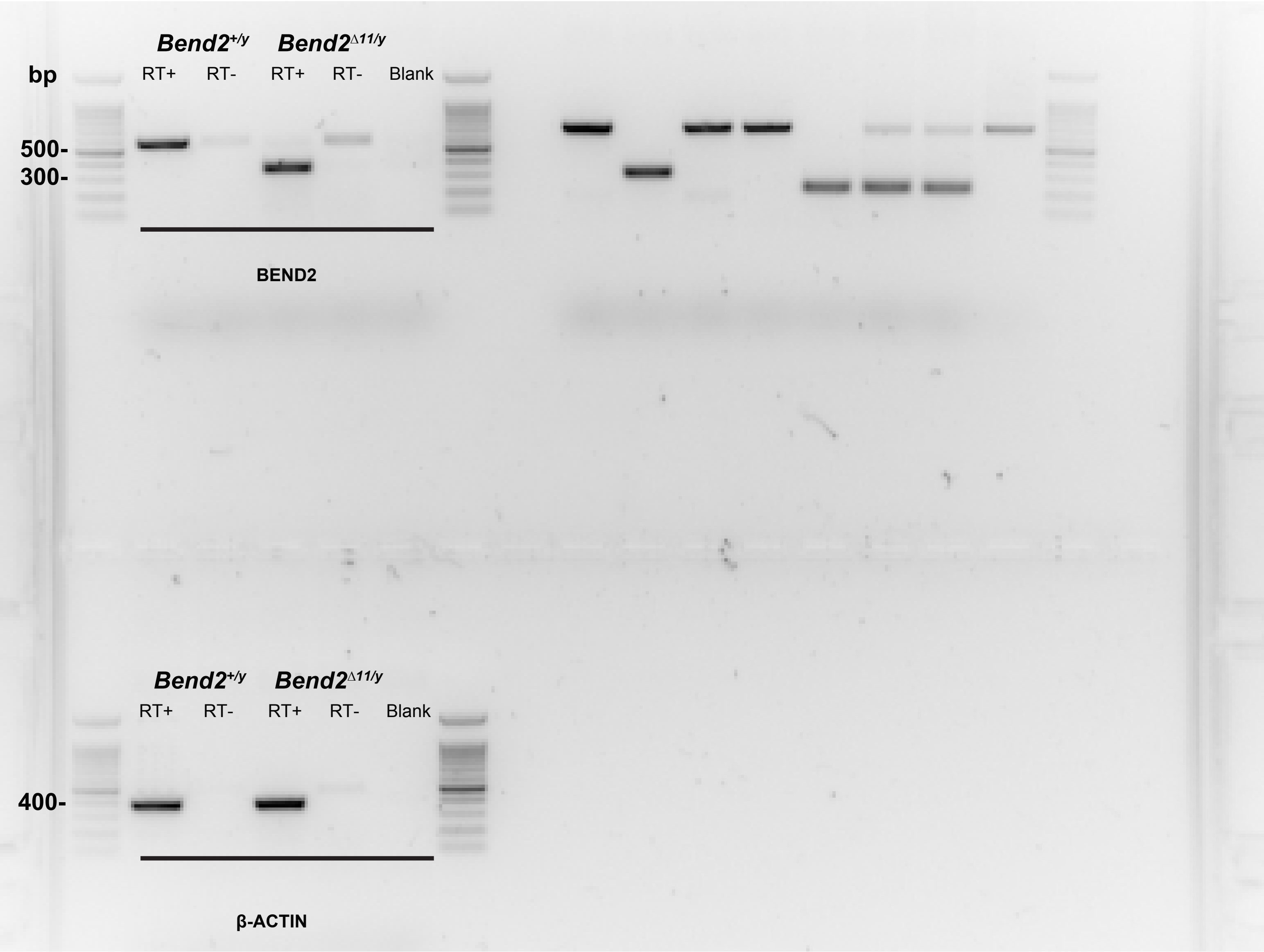

Figure 2, Source Data 2.
Original gel corresponding to Figure 2, panel B.
Left panel: BEND2 expression in control and knockout testis; β-ACTIN serves as a loading control.
Right panel: Expression of additional testis-expressed genes not included in Figure 2**.**
.

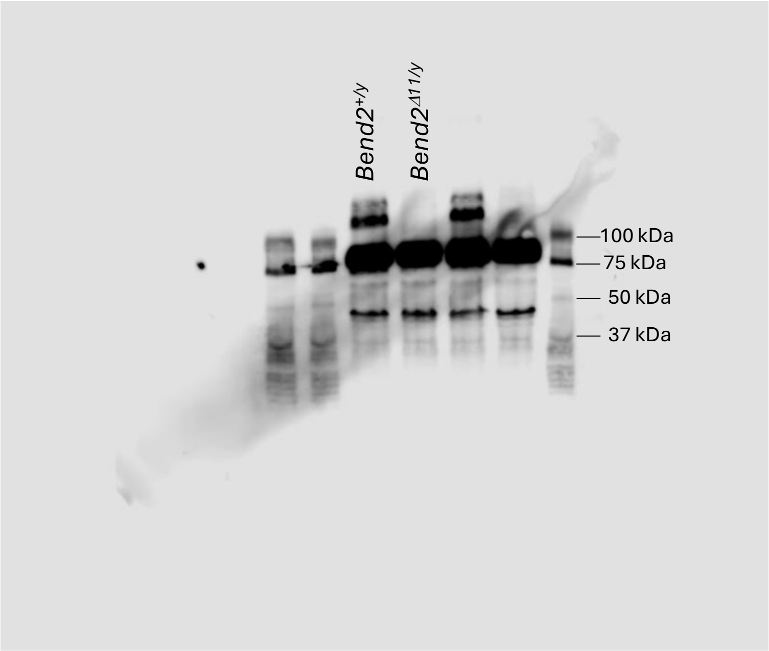

Figure 2, Source Data 2.
Original gel corresponding to Figure 2, panel C.
The labeled lanes show BEND2 expression in mouse adult testis. Bio-rad precision marker is employed
