# Supplementary material for: The full-length BEND2 protein is dispensable for spermatogenesis but required for setting the ovarian reserve in mice
Source: eLife. 2025 Aug 20;13:RP96052. doi: 10.7554/eLife.96052 (PMC12367297; doi:10.7554/eLife.96052)
Supplement: Figure 4—figure supplement 1—source data 2. [file elife-96052-fig4-figsupp1-data2.zip › Figure S2-source data 2/Figure S2 sourcedata2.pdf]

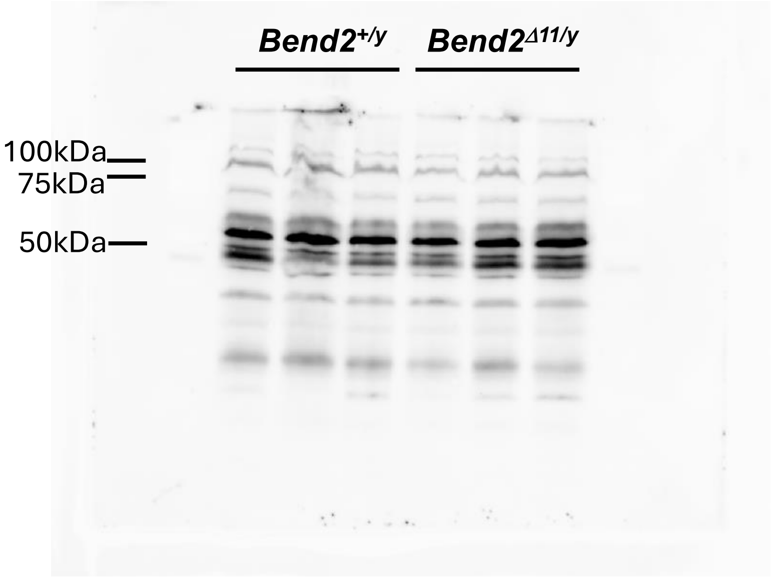

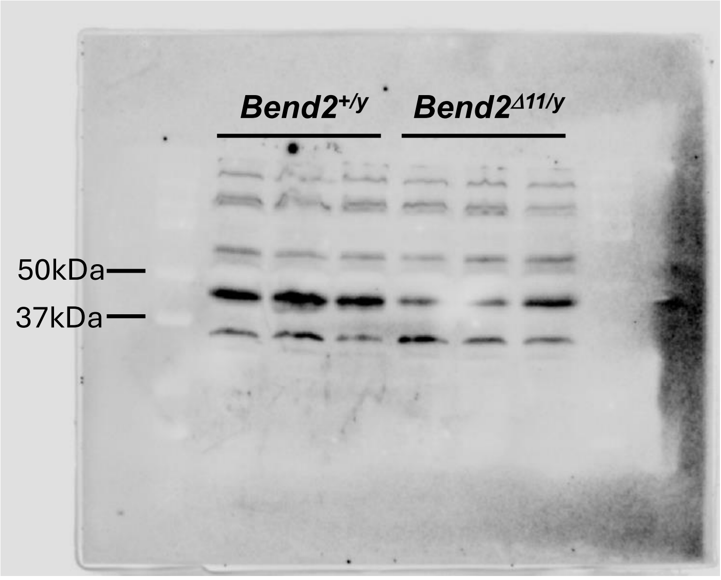

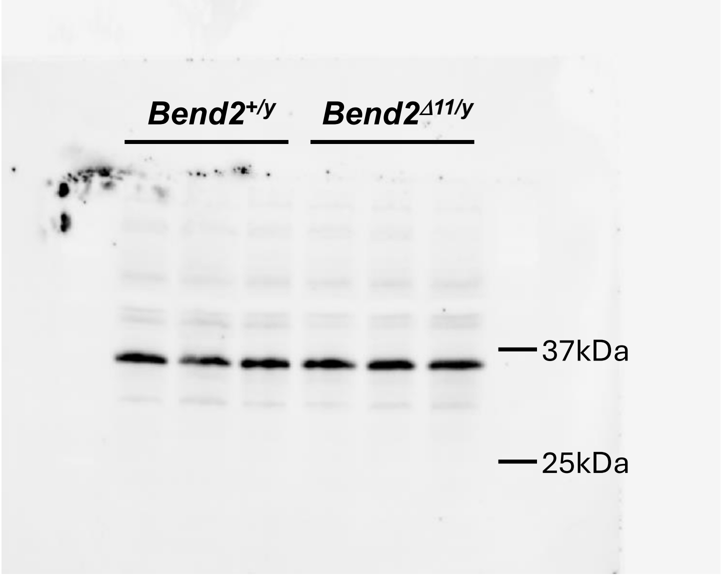

Figure S2, Source Data 2. Original gel corresponding to Figure S2, panel D.
Top panel: KU70 western blot; **Mid panel:** LINE-1 western blot; **Bottom panel:** GAPDH western blot.
