# Supplementary material for: The full-length BEND2 protein is dispensable for spermatogenesis but required for setting the ovarian reserve in mice
Source: eLife. 2025 Aug 20;13:RP96052. doi: 10.7554/eLife.96052 (PMC12367297; doi:10.7554/eLife.96052)
Supplement: Supplementary file 1. [file elife-96052-supp1.docx]

| Animal ID | Genotype | Age (weeks) | Testis weight (g) | Body weight (g) | TW/BW |
| --- | --- | --- | --- | --- | --- |
| 155 | *Bend2 ^+/y^* | 14 | 0,22 | 30,37 | 0,0072 |
| 156 | *Bend2 ^+/y^* | 14 | 0,22 | 31,32 | 0,0070 |
| 884 | *Bend2 ^+/y^* | 32 | 0,2648 | 50,66 | 0,0052 |
| 885 | *Bend2 ^+/y^* | 32 | 0,2232 | 47,03 | 0,0047 |
| 899 | *Bend2 ^+/y^* | 30 | 0,2321 | 40,61 | 0,0057 |
| 914 | *Bend2 ^+/y^* | 27 | 0,2515 | 35,02 | 0,0072 |
| 923 | *Bend2 ^+/y^* | 22 | 0,2418 | 32 | 0,0076 |
| 154 | *Bend2 ^∆11/y^* | 14 | 0,21 | 29,6 | 0,0071 |
| 157 | *Bend2 ^∆11/y^* | 14 | 0,19 | 27,16 | 0,0070 |
| 887 | *Bend2 ^∆11/y^* | 32 | 0,2016 | 41,18 | 0,0049 |
| 888 | *Bend2 ^∆11/y^* | 32 | 0,2472 | 43,88 | 0,0056 |
| 898 | *Bend2 ^∆11/y^* | 30 | 0,2151 | 43,9 | 0,0049 |
| 913 | *Bend2 ^∆11/y^* | 27 | 0,25 | 33,96 | 0,0074 |
| 922 | *Bend2 ^∆11/y^* | 22 | 0,2131 | 38,75 | 0,0055 |
